# Targeting 3-phosphoinositide-dependent protein kinase 1 by N-acetyl-cysteine through activation of peroxisome proliferators activated receptor alpha in human lung cancer cells, the role of p53 and p65

**DOI:** 10.1186/1756-9966-32-43

**Published:** 2013-07-18

**Authors:** Swei Sunny Hann, Fang Zheng, Shunyu Zhao

**Affiliations:** 1 Second Clinical Medical Collage, University of Guangzhou Traditional Chinese Medicine, Guangdong Academy of Traditional Chinese Medicine, Guangdong Provincial Hospital of Chinese Medicine, Room 421, 4th Floor, Scientific Research Building, Neihuan West Road No. 55, University City, Panyu District, Guangzhou, Guangdong Province 510120, P.R. China

**Keywords:** 3-phosphoinositide-dependent protein kinase 1, N-Acetyl-Cysteine, Peroxisome proliferators activated receptor alpha, Human non-small cell lung carcinoma cells, Nuclear factor-kappa B, p53

## Abstract

**Background:**

N-Acetyl-Cysteine (NAC), a natural sulfur-containing amino acid derivative, and peroxisome proliferators activated receptor alpha (PPARα) ligand have been shown to have anticancer properties. However, the mechanisms by which these agents inhibit human non-small cell lung carcinoma (NSCLC) cell growth have not been well elucidated.

**Methods:**

Small interfering RNAs (siRNAs) were used to knockdown 3-phosphoinositide-dependent protein kinase 1 (PDK1), PPARα, p65 and p53 genes; Western Blot was performed to detect the protein expression of PDK1, PPARα, p65 and p53; Cell viability and MTT assays were carried out to determine the cell proliferation; Transient transfection and Dual-Luciferase Reporter assays were used to transfect siRNAs or exogenous expression vectors, and to measure the gene promoter activity.

**Results:**

We showed that NAC inhibited NSCLC cell proliferation through reduction of PDK1 expression. NAC also induced the protein expression of PPARα. While PPARα ligand enhanced, PPARα antagonist and siRNA abrogated the effect of NAC on PDK1 promoter activity, protein expression and cell growth. Overexpression of PDK1 diminished the inhibitory effect of NAC on cell proliferation. NAC induced p53 and reduced p65 protein expression through activation of PPARα. Silencing of p53 and overexpression of p65 blocked the effect of NAC on PDK1 promoter activity and protein expression.

**Conclusion:**

Our results show that NAC inhibits PDK1 expression through PPARα-mediated induction of p53 and inhibition of p65 protein expression. PPARα ligand enhances the effect of NAC. This ultimately inhibits NSCLC cell growth. This study unveils a novel mechanism by which NAC in combination with PPARα ligand inhibits growth of human lung carcinoma cells.

## Background

Lung cancer continues to be the most frequent cancer-related cause of death throughout the world with a poor 5-year survival rate (< 15%) [1]. New approaches to the treatment and prevention of lung carcinoma depend on a better understanding of the cellular and molecular mechanisms that control tumor growth in the lung. N-Acetyl-Cysteine (NAC), a natural sulfur-containing amino acid derivative and a powerful antioxidant, has been shown to inhibit inflammatory responses, tumor progression [2,3]. However, the mechanisms by which NAC inhibits growth of human lung cancer cells have not been well characterized.

In an effort to explore the anti-tumor effects of NAC on potential targets, we turned our attention to 3-phosphoinositide-dependent protein kinase 1 (PDK1), a master regulator of signal cascades that are involved in suppression of apoptosis and promotion of tumor growth including lung cancer [4]. High expression of PDK1 has been detected in various invasive cancers [5]. Reduction of PDK1 by small interfering RNA (siRNA) in several cancer cells results in significant cell growth inhibition [6]. These observations suggest that PDK1 can be used as a potential target for cancer therapies.

Peroxisome proliferators activated receptor alpha (PPARα), a ligand-inducible nuclear transcription factor that has been implicated in the pathogenesis and treatment of tumor including lung cancer [7]. However, the exact role that PPARα signaling plays involved in non small cell lung carcinoma (NSCLC) biology and the mechanisms by which PPARα ligands suppress tumor cell growth have not been fully elucidated. A report showed that NAC could increase PPARα activity [8].

Herein, our results show that NAC inhibits expression of PDK1 expression through PPARα-mediated induction of p53 and inhibition of p65 protein expression.

## Methods

### Culture and chemicals

NSCLC cell lines H1650, A549, H1792, H2106, H460 and H358 were obtained from the American Type Culture Collection (Manassas, VA, USA), and were grown in RPMI-1640 medium supplemented with 10% FBS, HEPES buffer, 50 IU/mL penicillin/streptomycin, and 1 μg amphotericin. All cell lines have been tested and authenticated for absence of *Mycoplasma*, genotypes, drug response, and morphology in the Laboratory in May 2010 and April 2012. Polyclonal antibodies specific for PDK1, PPARα, p65, p50 and p53 were purchased from Cell Signaling Inc (Beverly, MA, USA). The Dual-Luciferase Reporter Assay kit was obtained from Promega (Shanghai, China). N-Acetyl-Cysteine (NAC), GW6471, fenofibrate and all other chemicals were purchased from Sigma Chemicals, Inc. (St. Louis, MO, USA) unless otherwise indicated.

### Treatment with PDK1, PPARα, p65 and p53 small interfering RNAs (siRNAs)

The siRNA human PDPK1 (EHU071261) was ordered from Sigma, PPARα siRNA (sc-36307), and p65 siRNA (sc-29410) were purchased from Santa Cruz Biotechnology. Signal Silence p53 siRNA (#6231) was ordered from Cell signaling. The control nonspecific siRNA oligonucleotide (D-001206-13-05) was purchased from Dharmacon, Inc. (Lafayette, CO, USA). For the transfection procedure, cells were grown to 60% confluence, and PDK1, PPARα and p53 siRNAs and control siRNA were transfected using the oligofectamine reagent (Invitrogen). Briefly, oligofectamine reagent was incubated with serum–free medium for 15 min. Subsequently, a mixture of respective siRNA was added. After incubation for 30 min at room temperature, the mixture was diluted with medium and added to each well. The final concentration of siRNAs in each well was 70–100 nM. After culturing for 30 h, cells were washed, resuspended in new culture media in the control or treated plates for an additional 24 or 48 h for the following experiments.

### Western blot analysis

Equal amounts of protein from whole cell lysates were solubilized in 2 × SDS-sample buffer, separated on SDS-polyacrylamide gels. The separated proteins were transferred onto nitrocellulose using a Bio-Rad Trans Blot semidry transfer apparatus for 1 h at 25 voltages, blocked with Blotto with 5% nonfat dry milk and 0.1% Tween 20 for overnight at 4 C, and washed with wash buffer. Blots were incubated with polyclonal antibodies against PDK1, PPARα, p53, p65 and p50 (1:1000) for overnight at 37 C, washed and incubated with a secondary antibody raised against rabbit IgG conjugated to horseradish peroxidase (1:15000, Sigma, Beijing, China) for 1 h at room temperature. The washed blots were transferred to freshly made ECL Prime (Pierce, Rockford, IL, USA) and exposed to X-ray film.

### Cell viability assay

NSCLC cells (10^5^ cells/well) were transfected with control, PDK1 or PPARα siRNAs for 30 h before exposing the cells to NAC for an additional 48 h in 96-well plates. In parallel experiments, cells were transfected with control or overexpression PDK1 vector obtained from Addgene [9]. Afterwards, the numbers of viable cells in culture were determined using The CellTiter-Glo Luminescent Cell Viability kit according to the manufacturer’s instructions (Promega, USA).

## MTT assay

Cell viability was analyzed by the MTT [3-(4, 5-dimethylthiazol-2-yl)-2, 5-diphenyl tetrazolium bromide] assay. Briefly, cells were seeded in 96-well plates at the density of 1.5 × 10^3^ cells/well and were cultured with NAC for up to 48 h, and then 10 μL of 10 mg/mL MTT solution was added to each well for an additional 4 h according to manufacturer instructions. (Promega, Shanghai, China). After centrifugation, 150 μL dimethyl sulfoxide was added to the precipitate and the absorbance of the enzyme was measured at 490 nm using an Microplate Reader (Bio-Rad, Hercules, CA, USA). Cell growth rates (average absorbance of each treated group and treated group) were then calculated. All experiments were performed in triplicate samples and repeated at least three times.

### Transient transfection assay

The original human PDK1 promoter construct was a gift from Dr. Michalik at the University of Lausanne and have been reported previously [10]. The PDK1 promoter construct contains approximately 1500 base pairs of the 5’ flanking region of the human PDK1 gene connected to the pGL2 basic luciferase reporter vector [10]. Briefly, NSCLC cells were seeded at a density of 5 × 10^5^ cells/well in 6-well dishes and grown to 50 –60% confluence. For each well, 2 μg of the above PDK1 plasmid DNA constructs, or overexpression of PDK1(pDONR223-PDPK1) [9], or p65 vectors (pCMV4 p65) [11] with 0.2 μg of the internal control phRL-TK Renilla Luciferase Reporter Vector were co-transfected into the cells with the oligofectamine reagent (Invitrogen). In separate experiments, cells were transfected with control or PDK1, PPARα and p53 siRNAs (70 nM each) for 32 h followed by exposed the cells to NAC for an additional 24 h. The preparation of cell extracts and measurement of luciferase activities were carried out using the Dual-Luciferase Reporter Kit according to recommendations by the manufacturer. Changes in firefly luciferase activity were calculated and plotted after normalization with changes in Renilla luciferase activity within the same sample.

### Statistical analysis

All experiments were repeated a minimum of three times. All data were expressed in mean ± SD. The data presented in some figures are from a representative experiment, which was qualitatively similar in the replicate experiments. Statistical significance was determined with Student’s *t* test (two-tailed) comparison between two groups of data set. Asterisks shown in the figures indicate significant differences of experimental groups in comparison with the corresponding control condition (P < 0.05).

## Results

### NAC inhibits NSCLC cell proliferation through reduction of PDK1 protein expression

**Figure 1:**
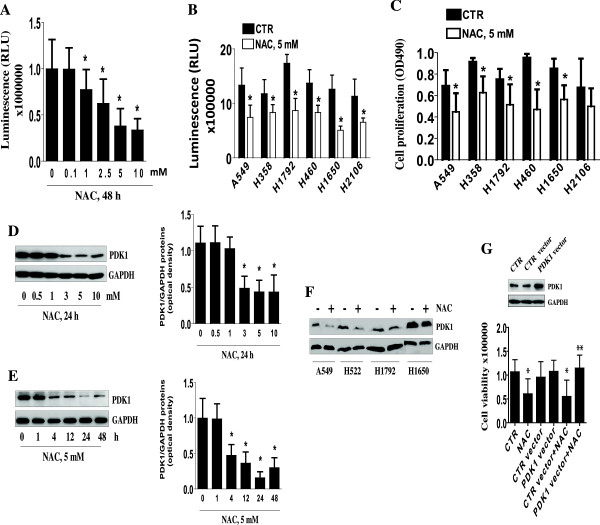
**NAC inhibits NSCLC cell proliferation through reduction of PDK1 protein expression. A-B,** A549 NSCLC cells exposed to increased concentrations of NAC for up to 48 h **(A)**, or NSCLC cell lines indicated were treated with NAC (5 mM) for up to 48 h **(B)**. Afterwards, cell proliferation was determined by Luminescent Cell Viability Assay. **C,** NSCLC cell lines indicated were treated with NAC (5 mM) for up to 48 h. Afterwards, cell proliferation was determined by MTT assays. Data are means ± SD from 3 separate experiments. *p < 0.01, compared with untreated cells (CTR). **D-E**, Cellular protein was isolated from A549 cells that were cultured with increased concentrations of NAC as indicated for 24 h (**D**) or cultured with NAC (5 mM) for the indicated time period (**E**) followed by Western blot analysis with antibodies against PDK1 protein. The bar graphs represent the mean ± SD of PDK1/GAPDH of at least three independent experiments. *indicates significant difference from untreated control (0). **F-G,** Several NSCLC cells as indicated were treated with NAC (5 mM) for 24 h followed by Western blot for detecting PDK1 protein. (**F**) or A549 cells were transfected with control or overexpression of PDK1 vectors for 24 h, followed by exposure of the cells to NAC for an additional 24 h. Afterwards, the luminescence of viable cells was detected using Cell Titer-Glo Luminescent Cell Viability Assay Kit. The upper panels represent protein levels of PDK1 by Western blot (**G**). All data were depicted as mean ± SD. *indicates significant difference as compared to the untreated control cells (CTR).

We first examined the effect of NAC on growth of lung carcinoma cells. A549 NSCLC cells exposed to increased concentrations of NAC for up to 48 h showed a significant decrease in cell proliferation with maximal reduction at 5 mM as determined by Luminescent Cell Viability Assay (Figure 1A). Similar results were observed in other NSCLC cell lines by this (Figure 1B) and as determined by MTT assays (Figure 1C).

We next determined the effect of NAC on PDK1 protein expression. Cells exposed to NAC resulted in significant decrease in PDK1 protein expression in a dose- and time-dependent manner with maximal induction noted at 5 mM at 24 h as determined by Western Blot (Figure 1D-E). NAC also reduced PDK1 protein expression in other NSCLC cell lines (Figure 1F). Overexpression of PDK1 has been reported to correlate with tumor progression [5]. We found that overexpression of PDK1 abrogated the effect of NAC on cell growth (Figure 1G, lower panel). Transfection with PDK1 expression vector was confirmed by Western blot (Figure 1G, upper panel). Together, these results suggest that NAC inhibits NSCLC cell growth through inhibition of PDK1.

### NAC induces protein expression of PPARα; blockade of PPARα abrogates the inhibitory effect of NAC on PDK1 protein expression and cell growth

**Figure 2:**
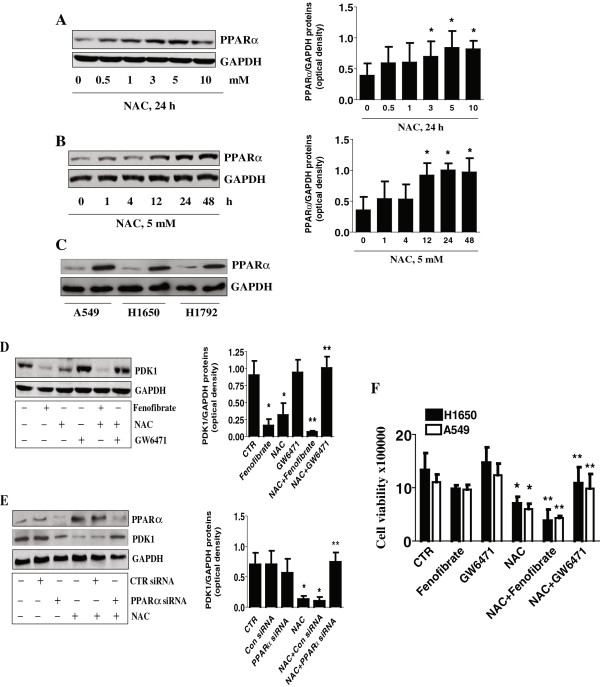
**NAC induces protein expression of PPARα; Blockade of PPARα abrogates the inhibitory effect of NAC on PDK1 expression and cell growth. A-B**, Cellular protein was isolated from A549 cells that were cultured with increased concentrations of NAC for 24 h **(A)** or cultured with NAC (5 mM) for the indicated time **(B)**, followed by Western blot analysis with antibodies against PPARα. The bar graphs represent the mean ± SD of PPARα/GAPDH of three independent experiments. *indicates significant difference from untreated control. **C**, Cellular protein was isolated from NSCLC cell lines that were cultured with NAC for 24 h followed by Western blot analysis with antibodies against PPARα protein. GAPDH used as loading control. CTR, indicates untreated cells. **D**, A549 cells were treated with GW6470 (20 μM) for 2 h before exposure of the cells to NAC (5 mM), Fenofibrate (10 μM) for an additional 24 h. Afterwards, Western blot analysis was performed to detect PDK1 protein. **E**, Cellular protein was isolated from A549 cells transfected with control or PPARα siRNA (100 nM each) for 30 h before exposure of the cells to NAC (5 mM) for an additional 24 h. Afterwards, Western blot analysis was performed to measure PPARα and PDK1 proteins. The bar graphs represent the mean ± SD of PDK1/GAPDH of three independent experiments. *indicates significant difference from untreated control. **indicates significance of combination treatment as compared with NAC alone (P < 0.05). **F**, A549 and H1650 cells were treated with GW6470 (20 μM) for 2 h before exposure of the cells to NAC (5 mM), Fenofibrate (10 μM) for an additional 48 h. Afterwards, the luminescence of viable cells was detected using Cell Viability Assay Kit. All data were depicted as mean ± SD. *indicates significant difference as compared to the untreated group (CTR).

We next determined the effect of NAC on PPARα protein levels. As shown in Figure 2A-B, NAC induced PPARα protein expression in a dose- and time-dependent manner with a maximal induction observed at 5 mM for 24 h. Similar results were also found in other NSCLC cell lines (Figure 2C). As we expected, blockade of PPARα with a chemical inhibitor, GW6471 [12], or the use of PPARα specific siRNA [12] abrogated the inhibitory effect of NAC on PDK1 protein expression (Figure 2D-E). Interestingly, the agonists of PPARα, fenofibrate, reduced PDK1 protein expression (Figure 2D). Finally, PPARα antagonist significantly overcame, while PPARα agonist enhanced the inhibitory effect of NAC on cell proliferation (Figure 2F).

### NAC reduces PDK1 promoter activity via PPARα

**Figure 3:**
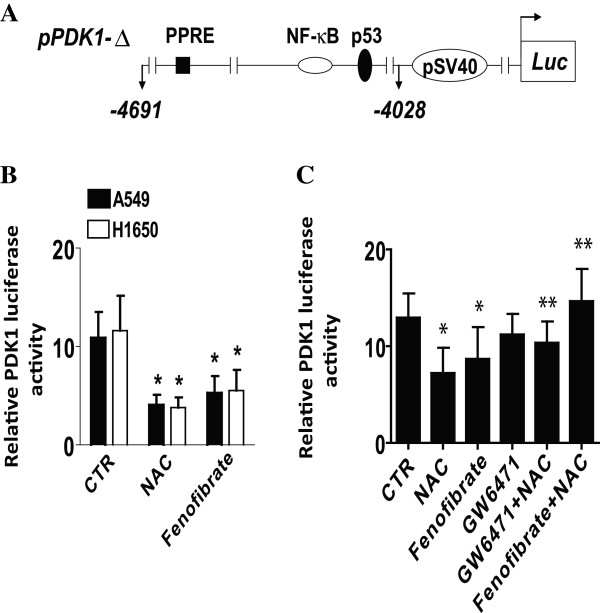
**NAC induces PDK1 promoter activity via PPARα. A,** The human PDK1 wild type promoter construct schematic is presented. These regions contain several transcription factor binding sites including c-myc, NF-κB, p53, among others. **B,** A549 and H1792 cells (1 × 10^5^ cells) were cotransfected with a wild type PDK1 promoter construct (shown in **A)** ligated to a luciferase reporter gene and an internal control phRL-TK Renilla Luciferase Vector for 24 h using the oligofectamine reagent (Invitrogen) according to the manufacturer’s instructions. After 24 h of incubation, cells were treated with NAC (5 mM) and Fenofibrate (10 μM) for an additional 24 h. **C**, A549 (1 × 10^5^ cells) were cotransfected with a wild type PDK1 promoter construct ligated to a luciferase reporter gene and an internal control phRL-TK Renilla Luciferase Vector for 24 h using the oligofectamine reagent. After 24 h of incubation, cells were treated with GW6470 (20 μM) for 2 h, followed by NAC (5 mM) and Fenofibrate (10 μM) for an additional 24 h. Afterwards, the ratio of firefly luciferase to renilla luciferase activity was quantified.

We also examined whether the effects of NAC on PDK1 expression occurred at the transcriptional level. As shown in Figure 3A, the PDK1 promoter contains multiple transcription factor binding sites including c-myc, nuclear factor-κB (NF-κB), p53, among others. We found that NSCLC cells transfected with wild-type PDK1 promoter-luciferase reporter construct showed decreased activity when exposed to NAC and fenofibrate (Figure 3B). GW7461 blocked the inhibitory effect of NAC and fenofibrate on PDK1 promoter activity suggesting a PPARα-dependent signaling in this process (Figure 3C).

### NAC induces p53 and reduces p63 protein expression through activation of PPARα; silencing of p53 and overexpression of p65 diminish the effect of NAC on PDK1 protein expression

**Figure 4:**
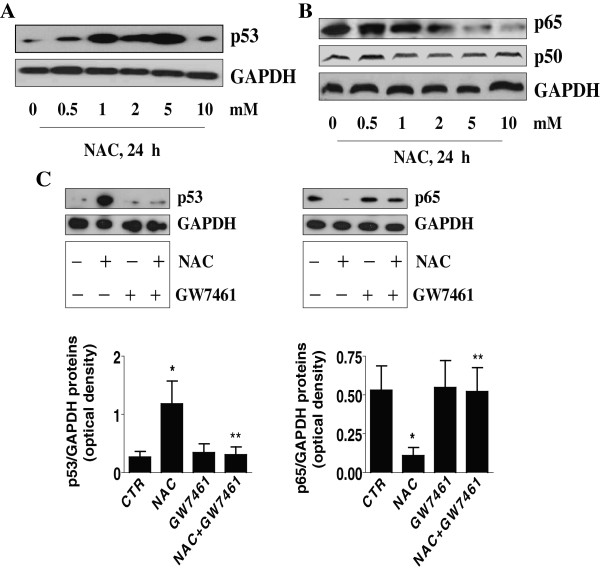
**NAC induces p53 and reduces p63 protein expression through activation of PPARα. A-B,** Cellular protein was isolated from A549 cells cultured with NAC (5 mM) for 24 h, followed by Western blot analysis with antibodies against p53, p50 and p65 proteins. **C**, A549 cells were treated with GW6470 (20 μM) for 2 h before exposure of the cells to NAC (5 mM) for an additional 24 h. Afterwards, Western blot analysis was performed using polyclonal antibodies against p53 and p65 protein. The bar graphs represent the mean ± SD of p53 or p65/GAPDH of at least three independent experiments. *indicates significance as compared with controls (CTR). **indicates significance of combination treatment as compared with NAC alone (*p* < 0.05).

**Figure 5:**
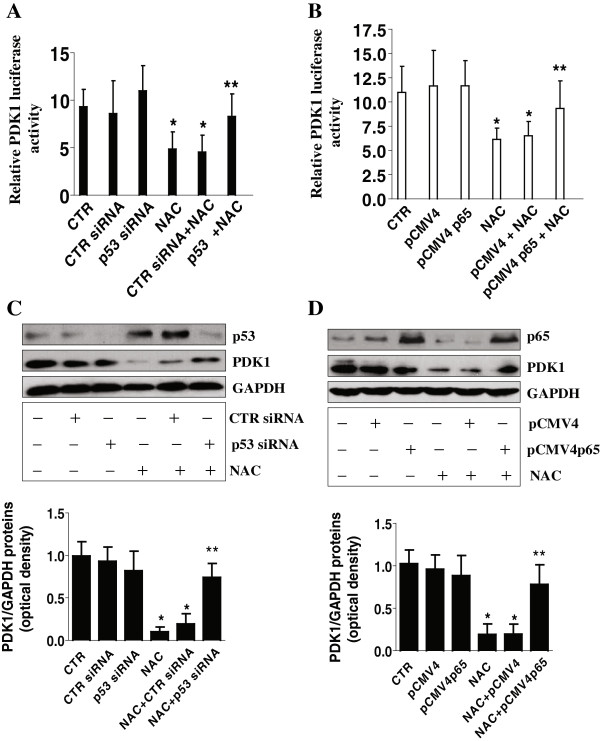
**Silencing of p53 and overexpression of p65 diminish the effect of NAC on PDK1 promoter activity and protein expression. A-B**, A549 cells (1 × 10^5^ cells) were cotransfected with a wild type PDK1 promoter construct and an internal control phRL-TK Renilla Luciferase Reporter Vector, and control or p53 siRNA (100 nM) for 40 h (**A)** or co-transfected with control or pCMV6 p65 expression vector (**B**) for 24 h, followed by NAC for an additional 24 h. Afterwards, luciferase assays were performed to detect PDK1 promoter activity. **C-D**, A549 cells were transfected with control or p53 siRNA (100 nM) for 40 h (**C**), and control or p65 overexpression vector for 24 h (**D**), followed by NAC for an additional 24 h. Afterwards, Western blot was performed to detect p53, p65 and PDK1 proteins. The bar graphs represent the mean ± SD of PDK1/GAPDH of at least three independent experiments. *indicates significance as compared with controls (CTR). **indicates significance of combination treatment as compared with NAC alone (*p* < 0.05).

In addition, we found that NAC increased protein expression of p53, a tumor suppressor (Figure 4A), while reducing NF-κB subunit, p65 protein expression in a dose-dependent manner (Figure 4B). Note that NAC had no effect on p50 protein (Figure 4B). Interestingly, GW7461 blocked the effect of NAC on p53 and p63 protein expression (Figure 4C). Furthermore, silencing of p53 or overexpression of p65 abrogated the effects of NAC on PDK1 promoter activity (Figure 5A-B) and protein expression (Figure 5C-D).

## Discussion

NAC, a common dietary supplement and an antioxidant membrane-permeable metal-binding compound, has been shown to inhibit inflammatory responses, tumor growth including lung cancer [13,14]. However, the mechanisms by which this reagent in control of NSCLC cell growth has not been well elucidated. We have found that NAC inhibited NSCLC cell proliferation through reduction of PDK1, a kinase and master regulator of a number of downstream signal cascades that are involved in suppression of apoptosis and promotion of tumor growth including lung cancer [4,15]. High expression of PDK1 has been detected in invasive cancers including lung [5] and inhibition of PDK1 in several cancer cells results in significant cell growth inhibition [6]. These observations suggest that PDK1 can be considered as a target for therapies. This result, together with the finding that exogenous PDK1 diminishes the inhibitory effect of NAC on cell growth, indicates an important role of targeting PDK1 in mediating the inhibitory effect of NAC on growth of NSCLC cells.

PPARα, a ligand-inducible nuclear transcription factor that has been implicated in the pathogenesis and treatment of tumor including lung cancer both *in vitro* and *in vivo*[7,16,17]. The exact role that PPARα signaling plays in NSCLC and the mechanisms by which PPARα ligands suppress tumor cell growth have not been fully elucidated. A report showed that NAC could increase PPARα activity [8]. Because of this, we will further test the role of PPARα and the effect of PPARα ligands on PDK1 expression. Our results showed that NAC increased protein expression of PPARα and the synergism of NAC and PPARα ligands on cell growth inhibition demonstrated an important role of this nuclear transcription factor in mediating the inhibitory effect of NAC on PDK1 expression and on NSCLC cell proliferation. Our result suggested that PPARα agonist could sensitize the effect of NAC on cell growth inhibition and also implied that NAC may act as a potential PPARα ligand. Consistent with this, one report demonstrated a synergistic effect of PPARα agonist and NAC in control of brain tumor cells [18].

Note that no report showed a link between PPARα ligand and PDK1 although PDK1 was reported to be a target gene of PPARσ/β [19], another isoforms of PPAR family, which strongly expressed in the majority of lung cancers, and activation of this isoform induced proliferation of lung cancer through pathways including activation of Akt phosphorylation correlated with up-regulation of PDK1 [20]. Note that the PDK1 promoter contains peroxisome proliferator responsive element (PPRE) [19], our data showed that PPARα ligand inhibited PDK1 promoter activity suggesting a distinct function of PPARα activation as compared to that of PPARσ/β. More studies are required to elucidate this.

Furthermore, our results indicated that NAC–mediated downregulation of PDK1 reflected inhibition of transactivation of the PDK1 gene and also demonstrated that NAC, through activation of PPARα, increased tumor suppressor, p53 and reduced p65, a subunit of NF-κB, which played important roles in mediating the effect of NAC on inhibition of PDK1 expression. This again suggested the characteristic of NAC acted as PPARα ligand. Silencing of p53 and overexprerssion of p65 blocked the effects of NAC on PDK1 expression further confirm the key roles of p53 and p65 in this process. P53 plays a critical role in tumor suppression mainly by inducing growth arrest, blocking angiogenesis and conferring the cancer cell sensitivity to chemoradiation [21]. Transcription factor NF-κB has been shown to regulate the expression of a number of genes that involve in many cellular processes such as inflammation and tumor growth [22]. Interestingly, the link of p53 in the regulation of glycolysis-dependent activation of NF-κB signaling in cancer has been reported [23]. However, the role of p53 and NF-κB in the direct regulation of PDK1 expression remains unknown. On the contrary, one study showed that overexpression of PDK1 resisted the apoptotic cell death caused by hypoxic injury and increased the expression of survival proteins, such as p53, in cultured rat cardiomyocytes [24]. Also, reports found that PDK1 plays a critical role by nucleating the T cell receptor-induced NF-κB activation pathway, which is important for T cell proliferation and activation during the adaptive immune response [25]. Together, these findings indicated that PDK1 was a critical regulator of tumor cell survival by modulating the p53 and NF-κB signaling pathways. NAC also had a direct or indirect effect on the regulation of p53 and NF-κB [26,27]. The activation of p53 has been shown to mediate the effects of NAC on prostate cancer cell growth [28]. One study reported that NAC inhibited growth of liver cancer cells through reducing the expression of NF-κB subunit p65 suggesting that NAC may be used for the treatment of liver tumor [29]. Thus, knockdown of p53 and overexpression of p65 abrogated the effect of NAC on PDK1 expression and cell proliferation highlighted the critical role of p53 and p65 in this process.

## Conclusion

In summary, our results show that NAC inhibits PDK1 expression through PPARα-mediated induction of p53 and reduction of p65 protein expression. Activation of PPARα enhances this process. This leads to inhibit NSCLC cell growth. This study unveils a novel mechanism by which NAC in combination with PPARα ligand inhibits growth of human lung carcinoma cells.

## Abbreviations

NAC: N-acetyl-cysteine; PPARα: Peroxisome proliferators activated receptor alpha; PPRE: Peroxisome proliferator response element; siRNAs: Small interfering RNAs; PDK1: 3-phosphoinositide-dependent protein kinase 1; NSCLC: Non-small cell lung carcinoma; MTT: 3-(4,5-dimethylthiazol-2-yl)-2,5-diphenyl tetrazolium bromide; NF-κB: Nuclear factor-kappa B.

## Competing interest

The authors declare that they have no competing interest.

## Authors’ contributions

SSH is fully responsible for the study design, performing experiments and drafting the manuscript. FZ carried out the MTT assays and statistical analysis. SYZ performed the densitometry, statistical analysis and participated in coordination manuscript. All authors read and approved the final manuscript.
